# Temperature Response of Soil Respiration in a Chinese Pine Plantation: Hysteresis and Seasonal vs. Diel *Q*_10_

**DOI:** 10.1371/journal.pone.0057858

**Published:** 2013-02-28

**Authors:** Xin Jia, Tianshan Zha, Bin Wu, Yuqing Zhang, Wenjing Chen, Xiaoping Wang, Haiqun Yu, Guimei He

**Affiliations:** 1 School of Soil and Water Conservation, Beijing Forestry University, Beijing, China; 2 Beijing Forestry Carbon Administration, Beijing, China; DOE Pacific Northwest National Laboratory, United States of America

## Abstract

Although the temperature response of soil respiration (*R_s_*) has been studied extensively, several issues remain unresolved, including hysteresis in the *R_s_*–temperature relationship and differences in the long- vs. short-term *R_s_* sensitivity to temperature. Progress on these issues will contribute to reduced uncertainties in carbon cycle modeling. We monitored soil CO_2_ efflux with an automated chamber system in a *Pinus tabulaeformis* plantation near Beijing throughout 2011. Soil temperature at 10-cm depth (*T_s_*) exerted a strong control over *R_s_*, with the annual temperature sensitivity (*Q*_10_) and basal rate at 10°C (*R_s_*_10_) being 2.76 and 1.40 µmol m^−2^ s^−1^, respectively. Both *R_s_* and short-term (i.e., daily) estimates of *R_s_*_10_ showed pronounced seasonal hysteresis with respect to *T_s_*, with the efflux in the second half of the year being larger than that early in the season for a given temperature. The hysteresis may be associated with the confounding effects of microbial population dynamics and/or litter input. As a result, all of the applied regression models failed to yield unbiased estimates of *R_s_* over the entire annual cycle. Lags between *R_s_* and *T_s_* were observed at the diel scale in the early and late growing season, but not in summer. The seasonality in these lags may be due to the use of a single *T_s_* measurement depth, which failed to represent seasonal changes in the depth of CO_2_ production. Daily estimates of *Q*_10_ averaged 2.04, smaller than the value obtained from the seasonal relationship. In addition, daily *Q*_10_ decreased with increasing *T_s_*, which may contribute feedback to the climate system under global warming scenarios. The use of a fixed, universal *Q*_10_ is considered adequate when modeling annual carbon budgets across large spatial extents. In contrast, a seasonally-varying, environmentally-controlled *Q*_10_ should be used when short-term accuracy is required.

## Introduction

A global effort is underway to mitigate anthropogenic climate change through afforestation/reforestation, in hope of sequestering carbon in plantation ecosystems. At the global scale, afforestation is occurring at 2.8 million ha yr^−1^ 1]. Understanding the environmental controls on carbon dynamics in new plantations is crucial for projecting future global carbon budget and climate scenarios, and could aid in assessing the effectiveness of carbon-oriented management practices in forestry.

Soil-surface CO_2_ efflux, commonly referred to as soil respiration (*R_s_*), constitutes a major source of carbon release to the atmosphere, and accounts for more than two-thirds of annual ecosystem respiration (*R_e_*) and one-half of gross ecosystem photosynthesis (*P_g_*) in temperate forests 2]. Aside from its large quantity, *R_s_* is exponentially related to soil temperature (*T_s_*) in most ecosystems 3,4]. Consequently, even subtle changes in climate (e.g., rising atmospheric temperature) could trigger significant changes in *R_s_*, markedly altering ecosystem carbon budgets. In turn, warming-induced increases in soil CO_2_ emissions could feed back to the climate system, although the intensity of climate–carbon cycle feedbacks remains an issue of debate 5]. Despite the large body of literature on the interactions between *R_s_* and climate change, the response of soil carbon processes to climatic factors (e.g., *T_s_* and soil moisture) is not well-known and remains a source of uncertainty in ecosystem carbon modeling 6,7].

Soil CO_2_ efflux is usually modeled as a simple function of *T_s_* (e.g., the classic *Q*_10_ function) at both diel and seasonal scales 2]. However, under field conditions the response of *R_s_* to *T_s_* is modulated by multiple factors at multiple temporal scales 8,9]. An increasing body of evidence indicates that forest *R_s_* is not adequately characterized by a simple function of *T_s_*, as other regulators (e.g., microbial dynamics, plant phenology and photosynthesis, soil water content and soil porosity) are able to confound the *R_s_*–*T_s_* relationship and lead to hysteresis (or phase lags) in the *R_s_*–*T_s_* relationship at multiple scales 8–10]. Hysteresis relationships provide information on the causality between two processes 9]. Detecting and interpreting the decoupling between *R_s_* and *T_s_* over timescales of hours to seasons can provide important insights into the mechanisms driving *R_s_* 9,10]. In addition, to accurately estimate carbon dynamics at multiple timescales in ecosystem carbon-cycle modeling, hysteresis relationships need to be explicitly considered 2,10]. The parameterization of *R_s_* and *R_e_* in carbon cycle models poses a major challenge when other factors confound the temperature response 7,11]. A recent synthesis reported that hysteresis in the *R_s_*–*T_s_* relationship is more common in forests than previously recognized 9].

Apart from hysteresis, confounding factors also cause a discrepancy between long-term (e.g., annual) and short-term (e.g., diel) temperature response parameters (e.g., *R_s_*_10_–the basal rate at 10°C; and *Q*_10_–the temperature sensitivity) 2,11]. The apparent annual *Q*_10_ may not reflect the true biotic temperature sensitivity if obscured by seasonally varying factors other than *T_s_* 11]. This is related to the ongoing debate on the use of a fixed (universal) vs. variable (environmentally-controlled) *Q*_10_ in carbon cycle modeling 7]. On the one hand, recent cross-site analyses point to a convergent sensitivity of respiration to temperature 7,12], negating previous conclusions that relate *Q*_10_ to climatic and substrate conditions 13,14]. Using FLUXNET data across 60 sites, Mahecha et al. 7] found that the apparent annual *Q*_10_ for *R_e_* decreased with increasing mean annual temperature, while short-term *Q*_10_, exempt from seasonally-confounding effects, converged to ∼1.4 across sites. In addition, a meta-analysis revealed that the seasonal *Q*_10_ for *R_s_* approximated 1.5 after excluding the confounding effects of vegetation seasonality 12]. On the other hand, single-site studies have reported large seasonal variation and temperature dependence of short-term unconfounded *Q*_10_ estimates for *R_s_* in forest ecosystems 2,6,11]. Therefore, comparing longer-term, apparent *Q*_10_ estimates of seasonal sensitivity with shorter-term estimates of daily sensitivity may provide new insights into the driving mechanisms of *R_s_* and *R_e_*, and shed light on model parameterization.

Detecting hysteresis at multiple timescales and resolving the aforementioned debate require long-term measurements of *R_s_* over both daily and seasonal cycles 15]. Recent studies have emphasized the use of automated chambers due to their ability to produce information about processes at fine temporal resolutions 16]. Continuous *R_s_* measurements in China's plantation forests are rare, despite the country's extensive efforts in afforestation (e.g., 8.43 million ha of new plantations from 2004 to 2008) 1]. The few existing studies were mostly based on measurements made at coarse intervals (e.g., days to weeks) 17,18], which are inadequate to fully unravel the dependency of *R_s_* on its controlling factors.

Using an automated chamber system, we monitored half-hourly values of *R_s_*, *T_s_* and soil volumetric water content (*VWC*) throughout 2011 in a Chinese pine (*Pinus tabulaeformis*) plantation at Badaling, about 50 km north of Beijing. Our objective was to quantify the seasonal and diel temperature responses of *R_s_*. We asked: (1) whether *R_s_* varies in-phase or out-of-phase with *T_s_* at diel and seasonal timescales; and (2) whether the apparent annual *Q*_10_ and *R_s_*_10_ are consistent with values derived at the diel timescale. Within-stand spatial uncertainty was also analyzed and briefly discussed. We paid special attention to the implications of these results for the parameterization of carbon cycle models.

## Materials and Methods

### 2.1. Ethics Statement

The study site is owned by Beijing Bureau of Forestry and Landscaping. The field work did not involve any endangered or protected species, and did not involve destructive sampling. Therefore, no specific permits were required for the described study.

### 2.2. Site description

The study site was a *P. tabulaeformis* plantation located in the Badaling Mountain region of Beijing (40°22.38'N, 115°56.65'E, 535 m a.s.l). The terrain is flat and uniform. The soil is of coarse-textured loess type, with phosphorous being the limiting nutrient for plant growth. The soil bulk density is 1.6 g cm^−3^. The plantation was a stand of 4-year-old *P. tabulaeformis* trees with a mean diameter at breast height (*DBH*) of 3.2±0.8 cm (± standard deviation, SD) and a mean height of 2.2±0.3 m in May, 2011. The stand density was 975 stems ha^−1^. The study site has no understory shrubs and only a sparse herbaceous cover (<10%).

The site is characterized by a temperate continental monsoon climate with hot and moist summers and cold and dry winters. Mean annual temperature (MAT) for 1985–2005 was 10.8°C, with highest and lowest mean monthly temperature of 26.9°C and −7.2°C in July and January, respectively (Meteorological Service of China). There were on average 160 frost-free days y^−1^. Mean annual precipitation (MAP) was 454 mm, 59% of which fell in July and August. Mean annual potential evapotranspiration was 1586 mm, about three times the precipitation. The study year (2011) was cooler and wetter than normal, with MAT and MAP being 9.2°C and 568 mm, respectively.

### 2.3. Field measurements

An automated chamber system was installed at the study site in November 2010 to make half-hourly measurements of *R_s_*. The system consisted of a LI-840 infrared gas analyzer (IRGA; LI-COR Inc., Lincoln, NE, USA), five custom-designed chambers, a CR1000 data logger (Campbell Scientific, Logan, UT, USA) and a rotary vane pump. Each chamber consisted of an alloy base and a moveable opaque dome. A pair of rotatable alloy arms connecting the dome and the base was promoted by a 12 V DC motor to open or close the chamber cap. When not in use, the chambers were kept open. The chamber base was placed over a fixed PVC collar which was 19 cm in diameter and 11 cm in height (inserted into the soil to a depth of about 7 cm). Collar insertion should have little impact on root dynamics because in this area most root biomass of *P. tabulaeformis* (>90%) is distributed at depths greater than 10 cm below the soil surface 19]. Rubber rings were used to seal the junctions among the chamber dome, base and collar. The tube connecting the chamber and the IRGA was about 15 m in length. The five chambers were randomly deployed in a 30-m diameter plot. A tube of 3 cm in length was mounted on the chamber as a vent to equalize the pressure inside and outside the chamber. Air temperature inside each chamber was measured using a type T thermocouple (Omega Engineering Inc., Stamford, CT, USA). The vegetation within collars was carefully removed one month before the start of measurements. Regrowth was minimal, and any regrowth was clipped regularly to avoid complication in the interpretation of the measurements.

The system measured soil CO_2_ efflux at half-hourly intervals. Five chambers, which shared a common IRGA through a multiplexer, were activated one at a time in each measurement cycle. Prior to closure, each chamber was purged with ambient air for 2 min to flush out the tubing. After closure, the air was circulated through the chamber and IRGA at a flow rate of 0.5 L min^−1^. The IRGA sampled CO_2_ ( µmol mol^−1^ moist air) and H_2_O (mmol mol^−1^ moist air) concentrations over a 2 min interval, and the data logger recorded the mole fractions at 2 s intervals. The data logger computed the rate of change in CO_2_ mixing ratio ( µmol mol^−1^ dry air) through linear regression of the CO_2_ mixing ratio against time (with a deadband of 10 s), and then calculated and stored the half-hourly rates of soil CO_2_ efflux.

Half-hourly *R_s_* ( µmol m^−2^ s^−1^) was computed as:
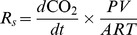
(1)


where *d*CO_2_/*dt* is the rate of change in CO_2_ mixing ratio over time. *P* is the atmospheric pressure (atm). *V* is the chamber volume (L), which is the sum of the aboveground collar volume and the chamber-top volume. *T* is the air temperature within the chamber (K), *A* the soil area within the collar (0.028 m^2^), and *R* the ideal gas constant (0.08206 L atm mol^−1^ K^−1^). The chamber-top volume was 2.8 L for all chambers. Collar volumes were calculated for each sampling location through multiplying the aboveground collar height by *A*.

Half-hourly *T_s_* and *VWC* at 10-cm depth were measured adjacent to each chamber. *VWC* was monitored with EC-5 soil moisture sensors (Decagon Devices Inc., Pullman, WA, USA) and *T_s_* was monitored with thermistor probes (Omega Engineering Inc., Stamford, CT, USA). Each month, three soil cores of 3 cm in diameter to a depth of 15 cm were collected close to each chamber and stored in plastic bags. The 5–15 cm depth section of the soil samples were taken to the laboratory, weighed, oven dried at 80°C to constant weight, and reweighed to determine the gravimetric water content. Bulk density was determined for the same soil samples. Automated *VWC* measurements were then calibrated against those derived from manual measurements on a monthly basis.

### 2.4. Data analysis

**Figure 1:**
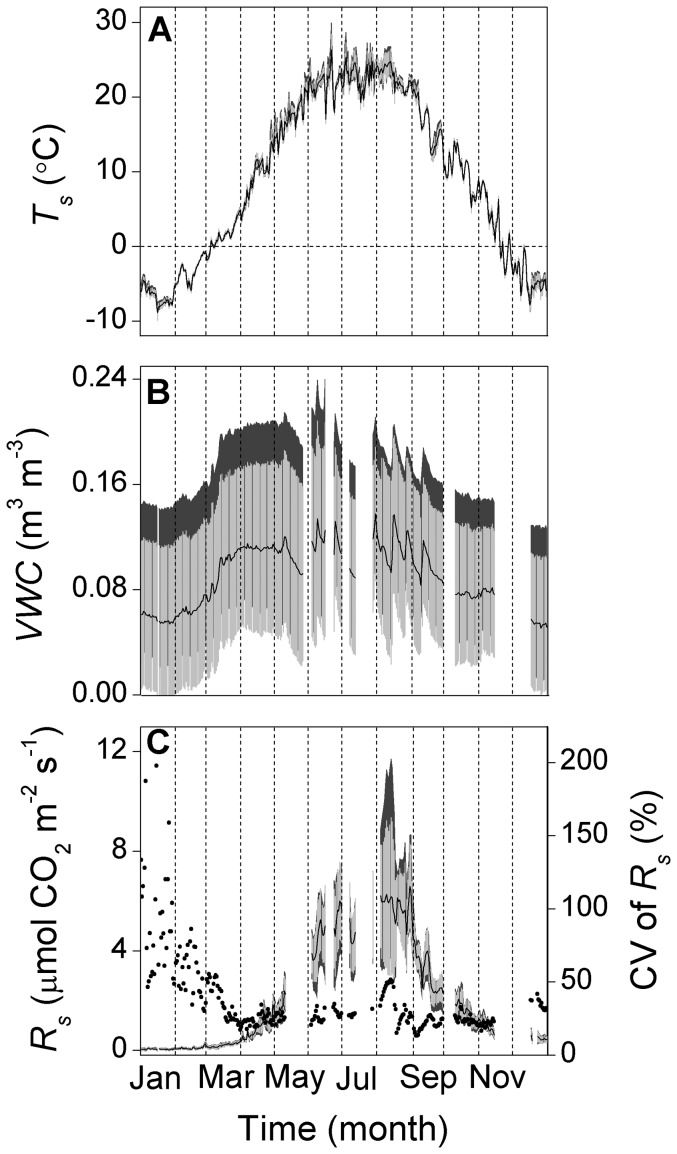
Soil temperature (*T_s_*) (A), volumetric water content (*VWC*) (B) and soil respiration (*R_s_*) (C). *T_s_* and *VWC* were monitored at 10-cm depth. Solid lines: mean across measurement locations; light grey: standard deviation among measurement locations; dark grey: range among measurement locations; black dots in (C): coefficient of variation (CV) for *R_s_*.

The half-hourly CO_2_ effluxes were screened as follows. Values outside the range of −5 to 20 µmol m^−2^ s^−1^ were considered abnormal and removed from the dataset. A mean ± 5SD criterion was then applied to monthly datasets to exclude outliers 1]. Instrument failure and quality control together resulted in 31% to 39% missing values for different chambers in 2011 (Fig. 1C). The remaining *R_s_* data spanned the annual cycles of both *T_s_* and *VWC*, allowing us to examine the relationships between *R_s_* and its regulating factors. In order to estimate annual *R_s_*, missing *T_s_* values were gap-filled using empirical relationships to half-hourly soil temperatures recorded at an eddy-covariance tower 30 m away. When the tower measurements were also missing, the mean diurnal variation (MDV) method 20] with weekly windows was used to fill gaps in *T_s_*.

The relationships between *R_s_* and *T_s_* were evaluated for both long-term (seasonal) and short-term (diel) timescales. The relationships were assessed for each sampling location separately, and also for the mean of the five chambers.

The long-term relationships were estimated based on daily mean values from complete annual cycle, using four common models: Exponential (*Q*_10_) 21], Arrhenius 21], Quadratic 1] and Logistic 1] (see Table 1 for the equations). Daily mean rather than half-hourly values were used to minimize noise caused by asynchrony at the diel scale. Recent studies have shown that daily values are more robust than hourly values for examining seasonal responses to temperature 22]. The *Q*_10_ model was also fit separately for each month. Root mean square error (RMSE) and the coefficient of determination (*R* ^2^) were used to evaluate model performance. RMSE and *R* ^2^ were compared among models using a bootstrap approach in which the dataset was sampled 2000 times, followed by one-way analyses of variance (ANOVA) and Tukey's HSD multiple comparisons.

**Table 1:** Parameters and statistics for the analysis of the dependence of daily mean soil respiration (*R*_s_) on soil temperature (*T*_s_).

Location	Model	Adj. *R* ^2^	RMSE	*R_s_*_10_/*R_s_*_283_	*Q*_10_	*E*_0_
Spatial mean	Exponential	0.925^d^	0.567^d^	1.40	2.76	
	Arrhenius	0.929^c^	0.550^c^	1.40		70.99
	Quadratic	0.941^b^	0.503^b^			
	Logistic	**0.948** ^a^	**0.473** ^a^		4.27	
Location #1	Exponential	0.818^d^	0.535^d^	1.05	2.30	
	Arrhenius	0.821^c^	0.537^c^	1.06		57.27
	Quadratic	0.850^b^	0.486^b^			
	Logistic	**0.854** ^a^	**0.486** ^a^		3.47	
Location #2	Exponential	0.866^a^	1.058^a^	1.52	3.57	
	Arrhenius	0.867^a^	1.054^a^	1.51		88.76
	Quadratic	0.859^b^	1.087^b^			
	Logistic	**0.868** ^a^	**1.052** ^a^		4.01	
Location #3	Exponential	0.900^d^	0.731^d^	1.66	2.56	
	Arrhenius	0.907^c^	0.706^c^	1.66		65.66
	Quadratic	0.929^b^	0.615^b^			
	Logistic	**0.945** ^a^	**0.544** ^a^		4.91	
Location #4	Exponential	0.899^d^	0.528^d^	1.09	2.61	
	Arrhenius	0.905^c^	0.514^c^	1.09		67.39
	Quadratic	0.918^b^	0.477^b^			
	Logistic	**0.929** ^a^	**0.443** ^a^		4.52	
Location #5	Exponential	0.958^c^	0.394^c^	1.32	3.39	
	Arrhenius	0.960^b^	0.386^b^	1.31		84.81
	Quadratic	0.954^d^	0.413^d^			
	Logistic	**0.963** ^a^	**0.371** ^a^		3.93	

Exponential: 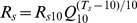; Arrhenius: 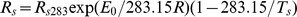; Quadratic: 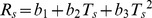; Logistic: 
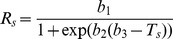
. *T_s_* was measured at the 10 cm depth. *R_s_*_10_ and *R_s_*_283_: basal rate of *R_s_* at 10°C, in units of µmol m^−2^ s^−1^; *Q*_10_: relative increase in *R_s_* for a 10°C increase in *T_s_*; *E*_0_: activation energy for *R_s_*, in units of KJ mol^−1^; *R*: universal gas constant (8.314 J mol^−1^ K^−1^); *b*_1_ through *b*_3_: fitted parameters. Adj. *R* ^2^: adjusted coefficient of determination; RMSE: root mean square error, in units of µmol m^−2^ s^−1^. Values in bold indicate best-fits according to Adj. *R* ^2^ and RMSE. Different letters following Adj. *R* ^2^ and RMSE indicate significant differences at the 0.05 level.

The short-term temperature response of *R_s_* was quantified using half-hourly data. A single model (the *Q*_10_ function) was applied to a four-day moving window with a one-day time step. To minimize the effects of rain pulses and maximize the robustness of parameter estimation, observations during rainfall or within two hours after rainfall were excluded from the analysis, and a minimum *R* ^2^ of 0.5 was required for a valid regression.

Cross-correlation analysis was used to detect hysteresis between *R_s_* and *T_s_* at both the seasonal and diel timescales 9,23], and to synchronize the values before the regression was performed. In the case of seasonal hysteresis, analysis of covariance (ANCOVA) was used to examine the difference in *R_s_* between the first (Jan–June) and second (July–Dec) half of the year, with *T_s_* as the covariate. Values of *R_s_* were log-transformed prior to ANCOVA to meet the assumptions of a normal distribution and linear correlation with the covariate. The range, SD and coefficient of variation (CV) were taken as indicators of spatial variability in *R_s_*, *R_s_*_10_ and *Q*_10_.

The monthly *Q*_10_ models were used to gap-fill daily mean *R_s_* and estimate annual total *R_s_*. The 95% confidence intervals (CI) for annual *R_s_* were estimated by bootstrapping, in which the gap-filled daily mean *R_s_* time series was sampled 2000 times. All analyses were processed in Matlab 7.11.0 (R2010b, The Mathworks Inc., Natick, MA, USA).

## Results

### 3.1. Seasonal pattern of *R_s_* and its temperature response

Daily mean *T_s_* was lowest on January 16th (−8.9°C), rose rapidly in February to June, remained high throughout summer (∼25°C), and decreased after mid August (Fig. 1A). Daily mean *VWC* averaged across locations was low in winter and high during the growing season, ranging from 0.05 to 0.14 m^3^ m^−3^ (Fig. 1B). Pulse dynamics in *VWC* were obvious from May through September (Fig. 1B). Daily mean *R_s_* averaged across locations showed strong but asymmetric seasonality over the year (Fig. 1C). Daily mean *R_s_* was lowest in January (<0.1 µmol m^−2^ s^−1^), did not show remarkable increases until March, peaked in August (>6.0 µmol m^−2^ s^−1^), and then decreased rapidly to ∼0.5 µmol m^−2^ s^−1^ at the end of the year. Cross-correlation analyses revealed that, although the correlation between daily mean *R_s_* and *T_s_* was highest at zero lag for all locations, the correlation coefficient was strongly asymmetric about the zero lag, with negative lags (*R_s_* lagging *T_s_*) reducing the correlation coefficient much more rapidly than positive lags.

Spatial variability in *R_s_* was substantial. The CV of daily *R_s_* among chambers varied between 10% and 50% from March to December (Fig. 1C), averaging 28%. The large CV in January and February was caused by the near-zero magnitude of *R_s_*. We did not find any evidence that the spatial variation in *R_s_* was related to *VWC* or the distance to trees.

**Figure 2:**
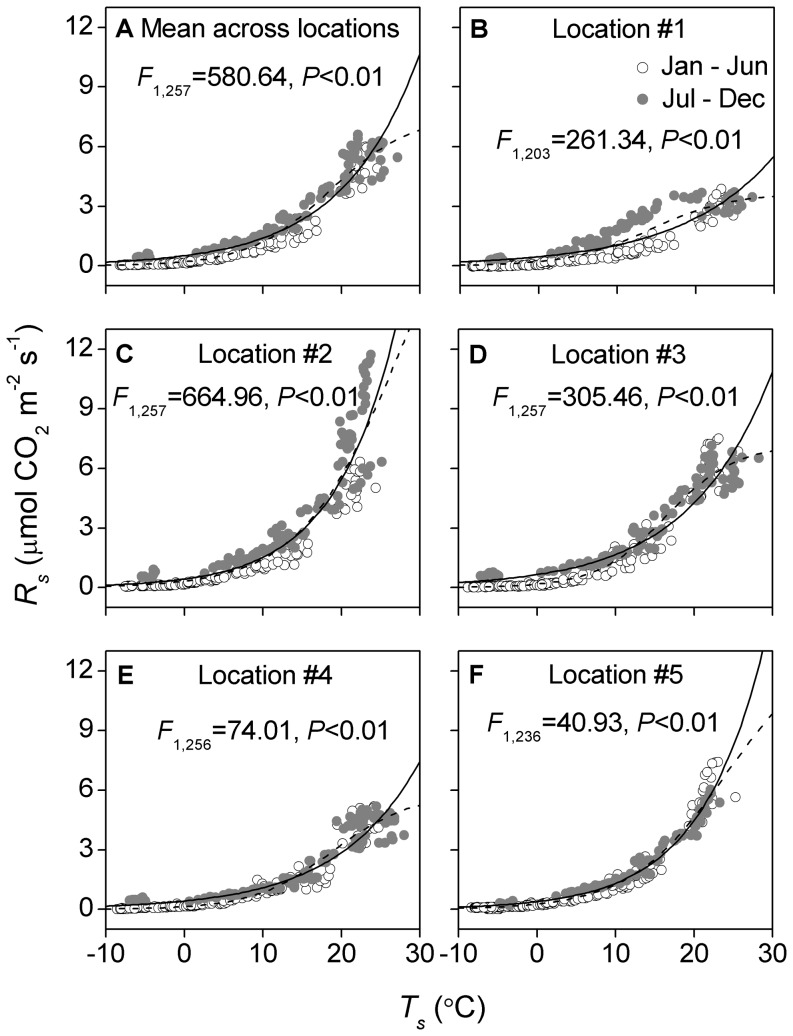
Relationships between daily mean soil respiration (*R_s_*) and soil temperature (*T_s_*). *T_s_* was monitored at 10-cm depth. Open circles are from January to June; closed circles are from July to December. The solid lines are fitted by a *Q*_10_ model; the dashed lines are fitted by a logistic model. *R_s_* is significantly different between the first and second half of the year when the *F*-test gives *P*<0.05.

**Figure 3:**
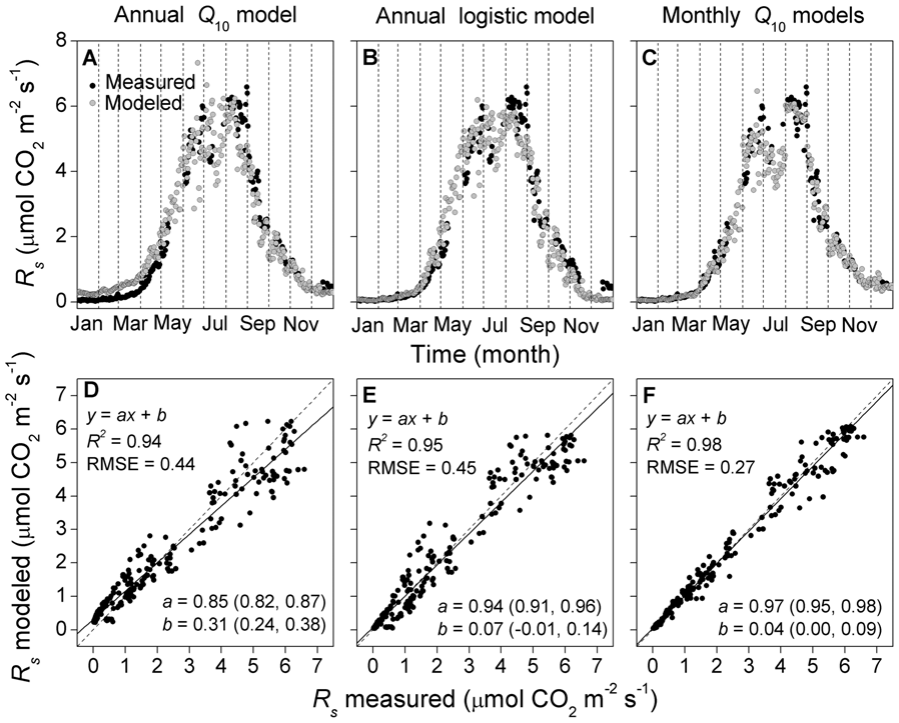
Comparisons between measured and modeled daily mean soil respiration (*R*_s_). Modeled *R_s_* values were derived from (A and D) an annual *Q*_10_ model, (B and E) an annual logistic model, or (C and F) monthly *Q*_10_ models. Values in parentheses in (D–F) represent 95% confidence intervals.

All four models of the seasonal *R_s_*–*T_s_* relationship performed well (Table 1). The three-parameter logistic model performed slightly better than the others, with consistently higher *R* ^2^ and lower RMSE. However, the annual model fits were unable to capture the pronounced seasonal hysteresis that was evident in the daily data, with *R_s_* in the second half of the season being larger than that in the first half at a given *T_s_* (Fig. 2). Significant seasonal hysteresis in the *R_s_*–*T_s_* relationship was observed for all sampling locations (and also for the spatial averages), with greater magnitudes for locations #1–3 than #4–5 (Fig. 2). As a result, the most commonly cited *Q*_10_ model and the best-fit logistic model both failed to yield unbiased *R_s_* estimates over the entire annual cycle. The *Q*_10_ model captured daily *R_s_* in autumn well, but overestimated *R_s_* in spring (Fig. 3A). In contrast, the logistic model underestimated daily *R_s_* in late autumn (Fig. 3B). The *R_s_modeled_* vs. *R_s_measured_* regression line significantly deviated from the 1 1 line according to the 95% CI for the slopes and intercepts (Fig. 3D, E). The estimation was greatly improved by fitting the *Q*_10_ model separately for each month (Fig. 3C). Monthly estimation enhanced the *R* ^2^ of the *R_s_modeled_* vs. *R_s_measured_* relationship, reduced the RMSE, and made the relationship closer to the 1 1 line (Fig. 3F). Temperature normalized *R_s_* (*R_sN_*, the ratio of observed to modeled values) for both the annual best-fit logistic model and monthly *Q*_10_ models were independent of *VWC* (results not shown).

The annual *Q*_10_ obtained from the exponential model was 2.76, varying from 2.30 to 3.57 across locations (Table 1). The estimated annual *R_s_* total, as calculated with monthly *Q*_10_ parameters and gap-filled *T_s_*, was 838 (758, 921) g C m^−2^. Across locations, annual *R_s_* varied from 538 (492, 585) to 1032 (920, 1146) g C m^−2^. The spatial uncertainty for annual *R_s_* was ±250 g C m^−2^, estimated as the 95% CI for *n* = 5 locations, assuming a *t* distribution with *n*−1 degrees of freedom and *α* = 0.05.

### 3.2. Diel temperature response of *R_s_*

**Figure 4:**
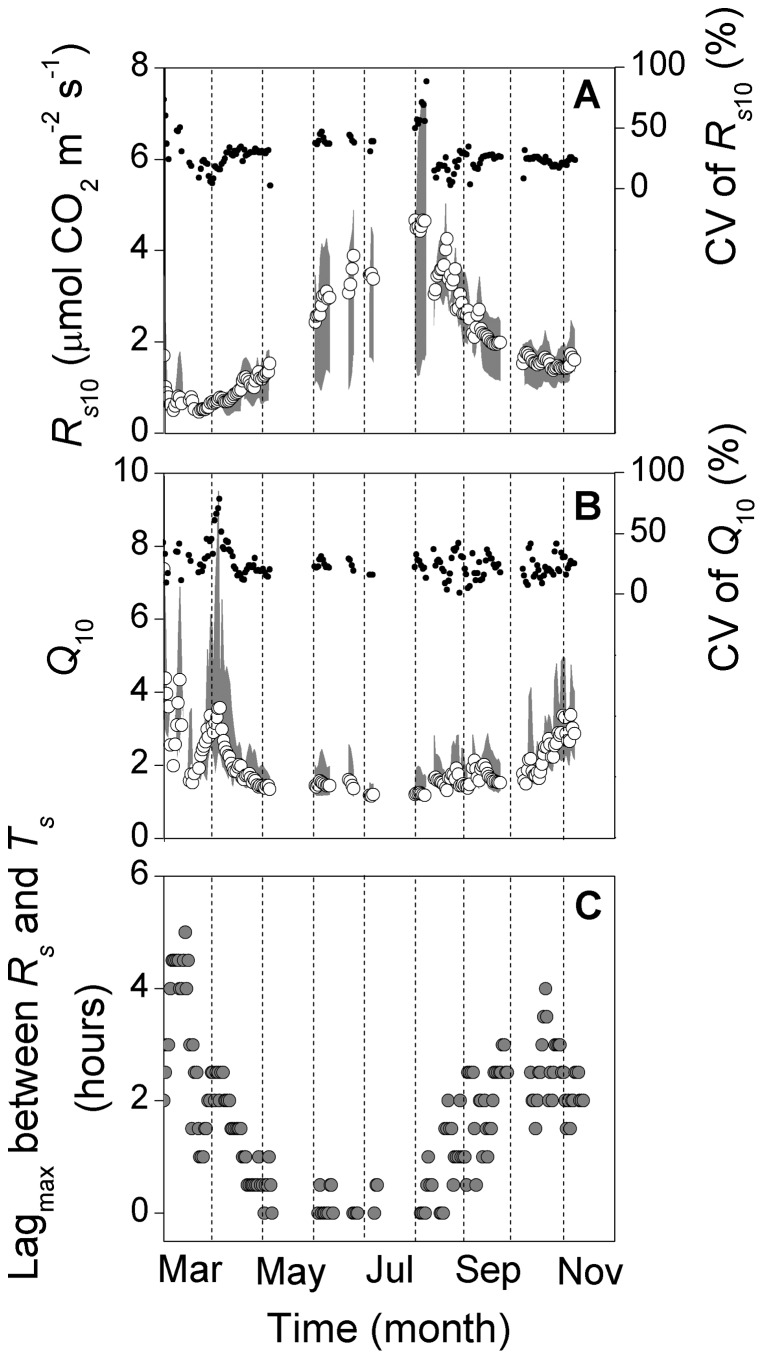
Daily *R_s_* _**10**_ **(A), daily** ***Q*** **_10_ (B) and diel lags (lag_max_) (C).** *R_s_*_10_ refers to the basal rate of soil respiration at 10°C. Lag_max_ indicates the temporal lag that maximizes the correlation between soil respiration (*R_s_*) and 10-cm soil temperature (*T_s_*) over the diel cycle. Circles in (A–C): mean across measurement locations; grey area in (A and B): range among measurement locations; black dots in (A and B): coefficients of variation (CV) for *R_s_*_10_ and *Q*_10_, respectively.

Both diel estimates of *R_s_*_10_ and *Q*_10_ showed strong seasonal trends (Fig. 4). Only the period from March to November is shown, as *R_s_* values were so small and *T*_s_ oscillated so weakly in winter that the regressions produced unreasonable parameter estimates. Mean *R_s_*_10_ across locations was <1.0 µmol m^−2^ s^−1^ in early March, increased throughout April to June, peaked in early August (∼4.5 µmol m^−2^ s^−1^), and then decreased to ∼1.50 µmol m^−2^ s^−1^ in November (Fig. 4A). *Q*_10_ was generally low in summer (1.5–2.0), but high at both ends of the growing season (2.0–4.0) (Fig. 4B). A peak in *Q*_10_ was evident between March and April.

The variability of *R_s_*_10_ and *Q*_10_ across locations can be quantified as functions of their magnitudes (robust regression with bisquare weights: *Range_R_s_*_10_ = 0.73 *R_s_*_10_–0.17, *R* ^2^ = 0.90; *Range*_*Q*_10_ = 0.82 *Q*_10_–0.47, *R* ^2^ = 0.78). Both daily *R_s_*_10_ and *Q*_10_ had CV values of between 0% and 50% for most time of the season, with high values of these parameters showing greater CV (Fig. 4A, B).

**Figure 5:**
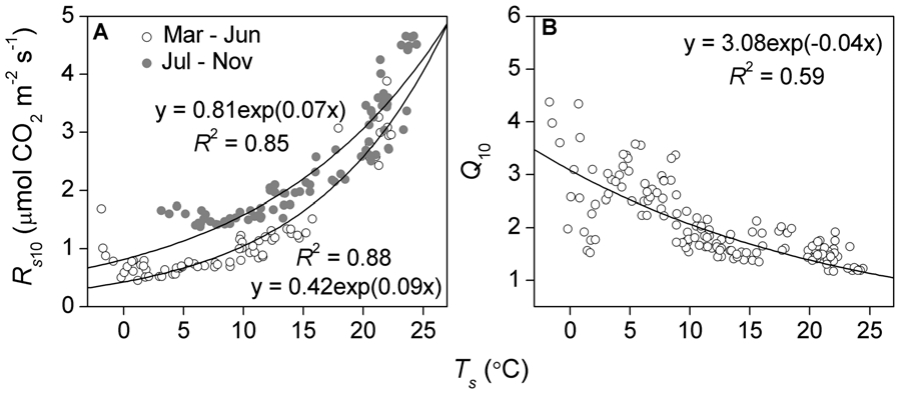
Relationships between soil temperature (*T_s_*) and (A) daily *R_s_* _**10**_ **and (B) daily** ***Q*** **_10_.** Open circles in (A) are from March to June, closed circles are from July to November.

Daily *R_s_*_10_ was positively correlated with *T_s_*, but with strong hysteresis (Fig. 5A). Fitting an exponential function of *T_s_* to the spring and autumn seasons separately explained more than 80% of the seasonal variation in *R_s_*_10_. Daily *Q*_10_ was negatively correlated with *T_s_* (Fig. 5B). An exponential function of *T_s_* accounted for 59% of the seasonal variation in *Q*_10_, with a decay rate constant of 0.04.

**Figure 6:**
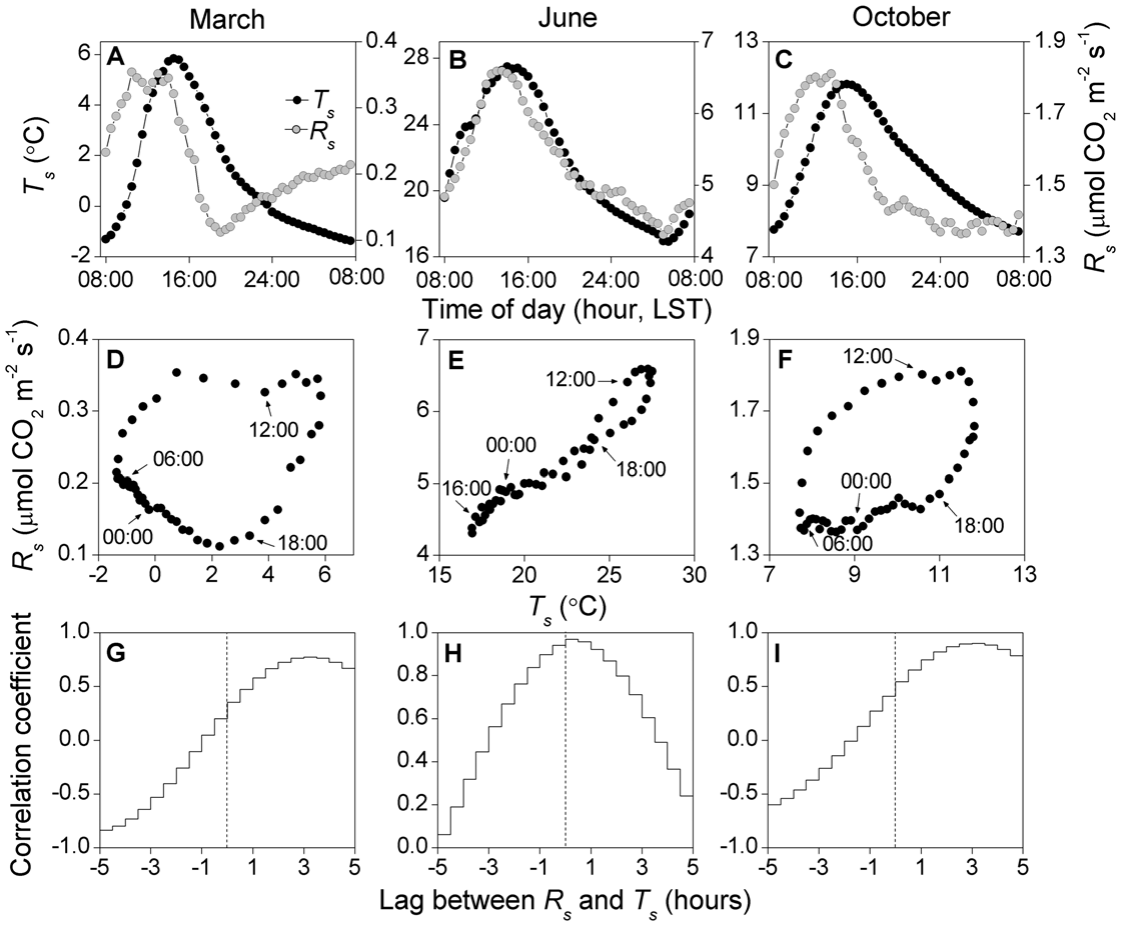
Diel soil respiration (*R_s_*) and temperature (*T_s_*) (A–C), diel *R_s_* vs. *T_s_* (D–I), their lag correlations (G–I). Mean values for March, June and October are shown. Grey circles in (A–C): *R_s_*; Black circles in (A–C): *T_s_*. *T_s_* was monitored at 10-cm depth. The dashed lines in (G–I) are reference lines for the zero lag.

The lag between diel oscillations in *R_s_* and *T*_s_ showed a strong seasonal pattern, with almost no lag in summer but lags up to five hours in the early and late growing season (Fig. 4C). In March and October, *T_s_* reached its daily minimum at 08:00 and peaked at around 15:00 (Fig. 6A, C). In March *R_s_* was out-of-phase with *T_s_*, reaching its daily maximum at 11:00–14:00 and daily minimum at 19:00. In October, *R_s_* was also out-of-phase with *T_s_*, peaking at around 12:00 and reaching a minimum at around 24:00. The lags in March and October led to hysteresis loops (Fig. 6D, F), and the correlation between *R_s_* and *T_s_* was strongest after lagging *R_s_* by three hours (Fig. 6G, I). In contrast, *R_s_* was in phase with *T_s_* in June (Fig. 6B, E), with the zero lag generating the highest correlation coefficient (Fig. 6H).

## Discussion

### 4.1. Temporal pattern of *R_s_* and hysteresis

Although the annual models fit the temperature response of *R_s_* reasonably well, they all failed to capture the seasonal dynamics of *R_s_* without bias over the annual cycle (Fig. 2, 3). This was due to the existence of seasonal hysteresis in the *R_s_*–*T_s_* relationship, which resulted in *R_s_* being greater in the second than the first half of the year for a given *T_s_* (Fig. 2). Hysteresis in the seasonal *R_s_*–*T_s_* relationship has been reported for various ecosystem types spanning a broad spectrum of climatic conditions, with the nature and magnitude of hysteresis varying across sites and vegetation types 8,9,24]. The decoupling of *R_s_* from *T_s_* is usually attributed to factors that confound the temperature effect. For example, Gaumont-Guay et al. 2] reported that a severe autumn drought caused seasonal hysteresis in the *R_s_*–*T_s_* relationship, leading to smaller *R_s_* in autumn than in spring for a given temperature. Biotic factors that may confound the *R_s_*–*T_s_* relationship include plant photosynthesis, root growth, litterfall dynamics and microbial dynamics 2,9,11]. These factors affect the timing and magnitude of different *R_s_* components, each of which can respond distinctly to *T_s_* 25,26]. The observed hysteresis in this study, i.e., with higher *R_s_* in the autumn than spring for a given *T_s_*, was in agreement with several previous studies 24,27,28]. The spring-autumn differences can result from increased soil microbial activity during late summer in response to the warming of deeper soil layers 2], or from the accumulation of fresh litter and/or respiring biomass (e.g. microbes and roots) as the season proceeded 4].

Soil moisture has been reported to regulate the seasonal temperature response of *R_s_*, e.g., *Q*_10_ decreases during drought 29]. However, we did not find any effect of soil *VWC* on *R_s_*. A lack of regulation of *R_s_* by soil moisture has also been reported for temperate and boreal coniferous forests 9,23]. The relatively low *VWC* values (0.05–0.14 m^3^ m^−3^), which reflect the high evapotranspiration, low soil water holding capacity and good drainage, may help explain the absence of *VWC* effect on *R_s_*. Moreover, soil moisture impacts on *R_s_* have been most commonly observed in arid or Mediterranean ecosystems, where hot and dry periods are common, during which *T_s_* and *VWC* are negatively correlated 9,29]. The temperate continental monsoon climate at our site features high summer precipitation (∼85% of the annual total fell from June to September in 2011), leading to a strong positive correlation between *T_s_* and *VWC* (*r* = 0.79; *P*<0.01) and providing adequate water for high rates of root and microbial metabolism. Despite the drought in winter, the concurrent low temperatures and thermal limitation may have cancelled the restriction of *R_s_* by low soil water (Fig. 1). Further investigation is needed to corroborate our conclusion on the role of *VWC* due to data gaps in summer (Fig. 1B, C).

We also observed diel lags in the *R_s_*–*T_s_* relationship (Fig. 4C, 6). Diurnal hysteresis has been quantified and modeled in various forest ecosystems, and was shown to either arise from the mismatch between the depth of temperature measurements and that of CO_2_ production, or the regulation of diurnal *R_s_* by the photosynthetic carbon supply 10,16]. More intriguingly, we found that the diurnal lag between *R_s_* and *T_s_* varied dramatically over the season; *R_s_* and *T_s_* were in-phase in summer, but *T_s_* lagged *R_s_* by about three hours in the early and late growing season (Fig. 4C, 6). Vargas et al. 16] also reported that the lag between hourly soil CO_2_ production and *T_s_* varied each day, showing that there is not a constant diel lag for each vegetation type. Seasonal changes in the diurnal lag as observed in our study may be the combined result of a varying depth of CO_2_ production over the season and a constant reference *T_s_* depth of 10 cm, i.e., with production at superficial layers in spring and autumn, and at deeper layers in summer. The primary depth of CO_2_ production may vary seasonally in association with changes in the relative contributions of autotrophic *vs.* heterotrophic respiration 23], as these components often occur at different depths (e.g., shallow litter and soil organic matter decomposition and deep root metabolism). The observed diel *R_s_*–*T_s_* lags in March and October were unlikely caused by diel variations in photosynthetic carbon supply because most studies demonstrate a higher autotrophic contribution to *R_s_* in the main growing season when plants are physiologically most active 23]. In addition, eddy-covariance measurements at our site revealed that ecosystem photosynthesis began in early May and ended in mid October, 2011 (unpublished data), and thus photosynthetic carbon supply was of little relevance to *R_s_* in March and October.

### 4.2. Long- vs. short-term temperature response

The short-term temperature response of *R_s_* (e.g. over the diel cycle) can deviate significantly from that for complete annual cycles because of seasonally-varying biophysical drives (e.g., root dynamics, plant photosynthesis) that confound the relationship of *R_s_* with temperature 2,4,11]. In this study, average daily *R_s_*_10_ (1.89) and *Q*_10_ (2.04) were higher and lower, respectively, than those obtained from the seasonal relationship (2.76 and 1.40 µmol m^−2^ s^−1^ respectively, Table 1 and Fig. 4A, B). High rates of plant photosynthesis and microbial metabolism in summer are supposed to enhance summer *R_s_* in addition to *T_s_*, causing a higher apparent annual *Q*_10_ 2,23,30]. In contrast, *Q*_10_ calculated from the short-term or high-frequency temperature response is exempt from seasonally confounding effects, and thus better reflects the biological sensitivity of respiration to temperature 6,7,11]. Diel *Q*_10_ exhibited large seasonal changes and decreased with increasing *T_s_* (Fig. 5B), which was consistent with many previous studies 2,6,11]. The reduction in *Q*_10_ with increasing *T_s_* may be associated with the transition from acclimation of enzymatic activity at low temperatures to limitation by substrate supply at high temperatures 2,31]. A peak of *Q*_10_ was obvious at the start of the growing season (Fig. 4B), and may reflect a jump in root activity and associated respiration 3]; some studies have demonstrated that autotrophic respiration is more sensitive than microbial respiration to temperature, with the qualification that these studies were based on seasonal rather than short-term responses 23,25,32].

A caveat should be noted when interpreting the dependence of short-term *Q*_10_ on temperature. Because the amplitude of *T_s_* oscillations dampens with depth in the soil profile, the decoupling of *T_s_* measurement depth from CO_2_ production depth may bias the estimation of temperature sensitivity 2,10]. The result will be an overestimation of *Q*_10_ when respiration occurs mostly above the temperature sensor (e.g., in the early and late growing season at our site), and an underestimation of *Q*_10_ when respiration occurs mostly below the temperature sensor. Therefore, the *Q*_10_–*T_s_* relationship in Fig. 5 might be partially explained by the dominance of shallow soil organic matter and litter decomposition (<10 cm) at both ends of the growing season when *T_s_* is low. Experiments incorporating multi-layer *T_s_* measurements or using the flux-gradient approach are needed to further assess the intrinsic relationship between *Q*_10_ and *T_s_*.

The large seasonal variation in the diel estimates of *R_s_*_10_ reported here was in accordance with existing results from forest studies 2,4], and was responsible for the discrepancy between the larger apparent annual *Q*_10_ and the smaller short-term *Q*_10_ estimates. The asymmetric seasonal pattern of *R_s_*_10_ resulted in a clear hysteresis relationship between *R_s_*_10_ and *T_s_* (Fig. 4A, 5A), which was similar to the mixed temperate forest study of Sampson et al. 4]. Instead of largely controlled by *T_s_* of *R_s_*, *R_s_*_10_ is usually an indicator of phenology, substrate supply, respiring biomass and the activity of roots and microbes 1,4]. The decoupling of daily *R_s_*_10_ from *T_s_* was responsible for the seasonal hysteresis relationships between *R_s_* and *T_s_* observed in this study (Fig. 2).

### 4.3. Spatial uncertainty of *R_s_*

Our results showed variations in the CV of *R_s_* among locations, ranging from 10% to 50% (Fig. 1C). These values are comparable to those found in an oak-grass savanna where the spatial heterogeneity in vegetation cover was much higher 33]. In a *Picea abies* stand, Buchmann 34] found that within-site variations of *R_s_* had a CV of 40%. Adachi et al. 35] reported CV of ∼40% for *R_s_* in two subtropical plantations. The mean annual *R_s_* of 838 g C m^−2^ from this study was greater than that found by Yu et al. 1] in a 50-year-old *Platycladus orientalis* plantation in Beijing (645 g C m^−2^). This discrepancy may arise from the different stand ages and the recent disturbance of the soil by afforestation at our site. Estimated annual *R_s_* at our site ranged from 538 to 1032 g C m^−2^, with a spatial uncertainty of ±250 g C m^−2^. Tang and Baldocchi 33] reported that the annual *R_s_* was 394 g C m^−2^ in the open area and 616 g C m^−2^ under trees in an oak-grass savanna. Davidson et al. 36] reported annual *R_s_* from a temperate mixed hardwood forest that ranged from 530 g C m^−2^ at the swamp site to 850 g C m^−2^ in a well-drained site. Therefore, the relatively uniform plantation we monitored exhibited *R_s_* that had comparable spatial variability to that in more heterogeneous stands, probably the consequence of high spatial variability in its biophysical factors 3,29]. The required number of measurement locations for estimating annual *R_s_* with error limits of 10% and 20% at our site was 45 and 11, respectively, calculated using the equation in 35].

Temperature response parameters also showed pronounced spatial variations (Fig. 2, 4A, B). The seasonal *Q*_10_ ranged spatially from 2.30 to 3.57; the daily *Q*_10_ showed CV values in the range of 0–50%. Xu and Qi 3] reported that the seasonal *Q*_10_ ranged spatially from 1.21 to 2.63 in a young ponderosa plantation in California, with a CV of larger than 20%. These results indicate that a spatially averaged *Q*_10_ may not be indicative of the sensitivity of *R_s_* to temperature in an ecosystem 3].

### 4.4. Conclusions and implications for carbon modeling

This study's main findings are: (1) despite a strong temperature control on *R_s_*, both *R_s_* and short-term estimates of *R_s_*_10_ showed pronounced seasonal hysteresis with respect to *T_s_* measured at 10-cm depth; (2) lags between *R_s_* and *T_s_* were observed at the diel timescale, but only in the early and late growing season; (3) the apparent annual *Q*_10_ (2.76) was larger than the mean daily *Q*_10_ (2.04), and daily *Q*_10_ decreased with increasing temperature. As detailed below, these findings have important implications for ecosystem carbon-cycle modeling.

Debate continues on the use of an invariant vs. biophysically-controlled temperature sensitivity to simulate respiration in carbon cycle models 6,7]. Some authors discovered that after ruling out seasonally confounding factors, convergent seasonal *Q*_10_ values (e.g., 1.4) emerged across sites spanning a diversity of climatic and vegetation conditions 7,12]. These studies negate previous conclusions relating *Q*_10_ to climate conditions 13,14] and argue for the use of a universal *Q*_10_ in modeling ecosystem respiration. In contrast, single-site continuous measurements have revealed large seasonal changes and environmental controls (e.g., soil temperature and moisture, substrate supply) on short-term unconfounded estimates of *Q*_10_ 2,6,11]. Our results add support to the latter finding, showing a clear dependence of daily *Q*_10_ on temperature over the growing season.

We propose, however, that the convergent seasonal *Q*_10_ and the seasonally-varying short-term *Q*_10_ are not necessarily in contradiction with each other, because they both exclude seasonally confounding effects. Both of them, therefore, reflect an unconfounded sensitivity to temperature, albeit at different temporal and spatial scales. The use of a constant vs. variable, environmentally-controlled *Q*_10_ in a carbon cycle model then becomes a matter of the scale on which carbon fluxes are simulated. A fixed annual *Q*_10_ is considered adequate when the model aims to predict annual carbon budgets at large spatial extents across climatic zones and ecosystem types 7]. In contrast, environmental controls on *Q*_10_ in a specific ecosystem should be taken into account when short-term accuracy is required to gain a mechanistic understanding of *R_s_* dynamics, to forecast the seasonality and diurnal course of *R_s_*, and to fill gaps in an *R*_s_ time series 6]. For example, eddy-covariance studies have demonstrated that using moving-window approaches (i.e., local fitting) to model the seasonality in the temperature sensitivity and thus the seasonal evolution of *R_e_* usually obtain better estimations than using a single, fixed annual function 20]. In addition, the use of variable, biophysically-controlled *Q*_10_ estimates has the potential to reproduce seasonal hysteresis in the *R_s_*–*T_s_* relationship, whereas a fixed annual parameter induces seasonal *R_s_* biases (Fig. 2, 3).

Another important factor in choosing the proper *Q*_10_ implementation is the level at which respiratory CO_2_ release is simulated. An ecosystem-specific empirical temperature response model which treats *R_s_* or *R_e_* as a composite flux (e.g., combining autotrophic and heterotrophic components) or as an emergent system behavior should adopt the apparent temperature response function because all effects on respiration, including those of confounding factors (e.g., plant phenology), have been implicitly incorporated into the model. In contrast, a process-based, bottom-up model, which explicitly simulates the mechanisms of different respiration components and their driving factors, should be parameterized with unconfounded short-term *Q*_10_ values for each component.

Lastly, previous studies 3] and our results imply that ecosystem carbon models should take into account the within-stand spatial uncertainty of temperature response parameters (e.g., as a function of their magnitudes, Fig. 4), rather than merely using a spatially deterministic value.
